# Efficacy of photobiomodulation in reducing pain and improving the quality of life in patients with idiopathic burning mouth syndrome. A systematic review and meta-analysis

**DOI:** 10.1007/s10103-022-03518-y

**Published:** 2022-02-05

**Authors:** Gisela Cristina Vianna Camolesi, Xabier Marichalar-Mendía, Maria Elena Padín-Iruegas, Juliana Cassol Spanemberg, Jose López-López, Andrés Blanco-Carrión, Pilar Gándara-Vila, Mercedes Gallas-Torreira, Mario Pérez-Sayáns

**Affiliations:** 1 Faculty of Medicine and Dentistry, University of Santiago de Compostela, Santiago de Compostela C.P, Entrerríos s/n, 15782 Galicia, Spain; 2 Oral Medicine, Oral Surgery and Implantology Unit (MedOralRes), Faculty of Medicine and Dentistry, University of Santiago de Compostela, Santiago de Compostela C.P, Entrerríos s/n, 15782 Galicia, Spain; 3 Nursing I Department, University of the Basque Country (UPV/EHU), C.P. 48940 Leioa, Bizkaia Spain; 4 Human Anatomy and Embryology Area, Department of Functional Biology and Health Sciences, Faculty of Physiotherapy, Pontevedra, Spain; 5 Oral Medicine and Public Health, Faculty of Dentistry, Universidad Fernando Pessoa-Canarias, Santa María de Guía, Calle de la Juventud s/n, C.P. 35450 Gran Canaria, Spain; 6 Department of Odontostomatology, Faculty of Medicine and Health Sciences, School of Dentistry, University of Barcelona//Oral Health and Masticatory System Group-IDIBELL, C.P. 08907 Barcelona, Cataluña Spain; 7 Health Research Institute of Santiago (IDIS) (ORALRES), Santiago de Compostela, Spain

**Keywords:** Primary burning mouth syndrome, Photobiomodulation therapy, Low lever laser therapy, Randomised clinical trials

## Abstract

Burning mouth syndrome is a chronic condition, which is characterised by a burning sensation or pain in the mucosa of the oral cavity. Treatment options include antidepressants, antipsychotics, anticonvulsants, analgesics, hormone replacement therapies and more recently photobiomodulation. This study aims to perform a systematic review with meta-analysis in order to determine the effect of photobiomodulation on pain relief and the oral health-related quality of life associated with this condition. A bibliographical search of the Pubmed, Embase, Web of Science and Scopus databases was conducted. Only randomised clinical trials were included. Pain and quality of life were calculated as mean difference and pooled at different treatment points (baseline = T0 and final time point = Tf) and laser modality. From a total of 103 records, 7 articles were retrieved for inclusion. PBM group had a greater decrease in pain than control group at Tf with a mean difference =  − 2.536 (IC 95% − 3.662 to − 1.410; *I*^2^ = 85.33%, *p* < 0.001). An improvement in oral health-related quality of life was observed in both groups, although this was more significant in the photobiomodulation group mean difference =  − 5.148 (IC 95% − 8.576 to − 1.719; *I*^2^ = 84.91%, *p* = 0.003). For the red laser, a greater improvement than infrared was observed, in pain, mean difference =  − 2.498 (IC 95% − 3.942 to − 1.053; *I*^2^ = 79.93%, *p* < 0.001), and in quality of life, mean difference =  − 8.144 (IC 95% − 12.082 to − 4.206; *I*^2^ = 64.22%, *p* = 0.027). Photobiomodulation, in particular, red laser protocols, resulted in improvement in pain and in quality of life of burning mouth syndrome patients.

## Introduction

The “International Headache Society” (IHS) defines burning mouth syndrome (BMS) as a chronic condition, which is characterised by a burning sensation or pain in the mucosa of the oral cavity. In clinical terms, the mucosa appears healthy without any obvious lesions, and this condition may be accompanied by xerostomia and/or dysgeusia. The sensation is recurrent on a daily basis, for more than 2 h/day, and for more than 3 months [1, 2]. The intensity of which ranges from moderate to severe, with it increasing throughout the day, although it tends to be absent at night. The tongue is the most affected area, followed by the lower lip and the hard palate [3, 4]. This sensation often persists for years and is the main cause of a decreased oral health-related quality of life (OHRQL) in patients with BMS [5].

Its prevalence is low, affecting between 0.1 and 3.7% of the general population [6], affects more women (1.15%) than it does men (0.38%), predominantly perimenopausal and/or postmenopausal women [7–9]. For some time, there have been indications that BMS affects people with personalities that are susceptible to anxiety and depression [6, 10, 11] more than those who do not experience such issues. It is a complex disease, and several pathophysiological mechanisms have been described that explain the condition [12, 13] of which the following are particularly worth mentioning: (i) alteration in dopaminergic transmission at a central level. In fact, the dopaminergic blink reflex is exaggerated in some patients with BMS [14]. (ii) Some type of peripheral neuropathy of the cranial nerves [14] given that neurophysiological and neuropathological studies have shown a loss of small diameter nerve fibres in the lingual epithelium [15], resulting to the depletion of neuroprotective steroids that alter the brain network related to mood and pain modulation [4, 16]. Which could explain the thermal and painful sensitivity in the tongue in these patients, as well as an increase in certain taste detection thresholds [17]. Some recent studies have suggested that, although not clinically visible, inflammation is the cause of the sensation of pain, with this being linked to cytokine actions [13]. Other studies have indicated that a genetic variation of the dopamine D2 receptor contributes to the sensation of pain [18]; nonetheless, mounting evidence has indicated the presence of hormonal, psychosocial, genetic and/or neuropathic causative factors [4], and, to date, final common consensus is yet to be reached.

Burning symptoms not attributable to local or systemic causes are currently considered as primary or idiopathic/essential BMS [1, 4, 19]. In order to effectively manage the treatment of these patients, their full medical, clinical, and dental history must be taken and the appropriate clinical and laboratory examinations must also be performed.

Drug treatments include antidepressants, antipsychotics, anticonvulsants, analgesics and hormone replacement therapies. Clonazepam is the most widely used and studied drug [20], and its efficacy has been demonstrated in recent meta-analysis [21]. Other prescribed drugs include alpha-lipoic acid (ALA), gabapentin, capsaicin and tricyclic antidepressants (TCAs) [22, 23]. Although a wide range of medications have been administered, these are not consistently effective for the majority of patients with BMS, and less than half of patients reported symptom relief following the administration of neuropathic drug therapies. Likewise, the multiple side effects make it impossible for patients to maintain long-term treatment fidelity [24].

In recent years, the use of laser biostimulation has been proposed for the treatment of chronic and acute pain [25], and its use for patients with BMS was first described in 2010 in a pilot study however indicated that it would be necessary to further studies with a more number of cases that are mandatory to obtain statistically significant results and versus control study is necessary [26]. Photobiomodulation (PBM) is a therapy that uses light, whether LED, red or infrared, to obtain beneficial effects on cells and tissues. It has an analgesic, anti-inflammatory and biological stimulation effect, resulting in improved pain relief and tissue healing [1, 16, 27].

Current BMS management focuses on the reduction of pain and the elimination of concomitant symptoms. Another important measure that must be taken into account when assessing the impact of BMS is the OHRQL, which shows the impact that severe pain can have on the patients’ well-being and emotional state.

This study aims to perform a systematic review and meta-analysis in order to determine the effect of PBM on pain relief and the OHRQL associated with primary BMS.

## Material and method

### Protocol and registration

**Fig. 1:**
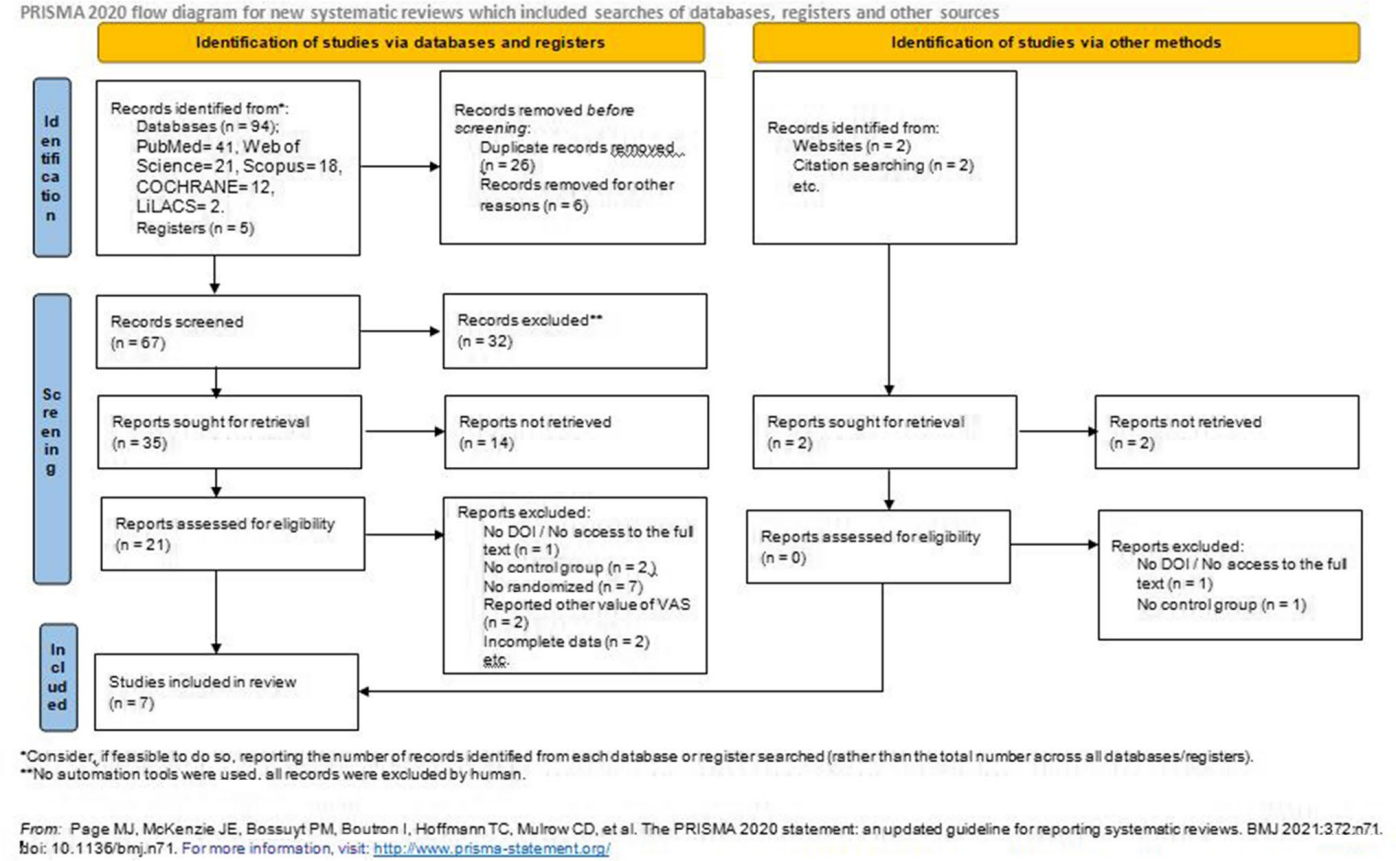
PRISMA flow; search strategy and selection of included articles

This review is an update of the review already recorded in PROSPERO (Ref. CRD42016048914), and it has been performed following the PRISMA guidelines [28] and according to the PICO method [29]: BMS patients (P = patient); PBM treatment with laser (I = intervention); laser off, or drug (C = comparison); remission of symptoms and improvement of OHRQL (O = outcome) (Fig. 1).

### Eligibility criteria, data sources and research

A bibliographic search was conducted using the following keywords: “burning mouth syndrome, photobiomodulation, LLLT, treatment” in PubMed, Web of Science, Scopus, LiLACS, OVID, EMBASE, Cochrane Library, Clinical Trials, the five regional bibliographic databases of the WHO (AIM, LILACS, IMEMR, IMSEAR, WPRIM), in order to identify relevant studies which compared the use of photobiomodulation in BMS patients to those forming a control group, describing all the types of interventions (Table 1). The search covered the first records found in the database right through to May 2021. Where necessary, the authors of the studies were contacted in order to identify missing information or data.

**Table 1:** Search criteria

Database	Keywords	Results
PubMed	burning mouth syndrome AND lllt AND photobiomodulation AND treatment (((burning mouth syndrome) AND (treatment)) AND (lllt)	009 032
Web of Science	burning mouth syndrome AND lllt AND photobiomodulation AND treatment (((burning mouth syndrome) AND (treatment)) AND (lllt)	004 017
Scopus	(((burning mouth syndrome) AND (lllt)) AND (photobiomodulation)) AND (treatment) (burning mouth syndrome) AND (treatment) AND (lllt)	002 016
LiLACS	“SINDROME DE BOCA ARDIENTE” and “Laser”	002
COCHRANE	burning mouth syndrome AND lllt AND photobiomodulation AND treatment burning mouth syndrome AND treatment and lllt	002 010

### Inclusion criteria

Articles that addressed randomised clinical trials, which included a well-defined control group in any language: patients who presented with primary BMS, patients who reported symptoms of burning or pain in the oral mucosa for at least 3 months. In the case in which other systemic diseases were present, it was required for these to be controlled.

### Exclusion criteria

Articles for which the abstract and/or full text were not available: studies with insufficient data; in vitro or animal studies; case reports or series, letters to the editor and/or editorials, literature reviews, books or book chapters, indexes and abstracts or dissertations, monographs and abstracts presented at scientific events; patients who had previously undergone radio and/or chemotherapy of the head and neck. With regard to the pain assessment, articles in which the visual analogue scale (VAS) or numerical rating scale (NRS) score was not between 0 and 10 (where 0 is no pain and 10 is excruciating pain), and/or articles that did not report “mean” and “SD” values, referring to the start and/or end of the treatment for VAS/NRS, and/or studies in which the OHRQL questionnaire used was not the Oral Health Impact Profile (OHIP). Studies with secondary BMS patients, whose condition was the result of organic causes, such as biological factors, that is to say, the presence of certain bacteria or fungi that have a direct irritant effect on the oral mucosa that could trigger burning symptoms, or systemic factors such as Sjögren’s syndrome, diabetes or if the medication used causes oral burning.

### Study selection

Two independent researchers, MPS and GCVC, selected the studies in a two-round process. The first round included an extensive analysis of the titles and abstracts of all of the articles that were obtained in the search. Studies that were unrelated to the topic of interest, that is to say texts that did not address the treatment of BMS patients with PBM, were eliminated. Titles and abstracts that met the criteria, but for which the abstracts were not available, were subsequently analysed in the second round. In the second round, all of the eligible studies were examined in full text before a decision was made as to whether or not the eligibility criteria had been met. The references included in the eligible articles were carefully screened in order to verify any studies that had not been detected through the main search strategy. The excluded studies were recorded separately, indicating the reasons for their exclusion.

### Data collection process

The full articles were read in order to determine whether the inclusion criteria had been met, and both researchers (MPS and GCVC) collected the data (in duplicate) to prevent any measurement bias. A third researcher, JLL, acted as a mediator in the case of any discrepancies, or in the case in which an agreement could not be reached. Cronbach’s alpha was calculated in order to determine the inter-researcher agreement, with a score of 0.93 [30].

### Study variables

The following data was extracted from each study: first author, year of publication, country of origin, sample size and details such as gender, mean age, the type of intervention in the control group, type of laser and amount of energy applied, total number of sessions, total duration of the treatment, time of completed treatment segment, request for analyses and the method used to assess the results (pain relief by VAS/NVA (0–10) and quality of life by OHIP-14 questionnaire) (Table 2).

**Table 2:** Detailed descriptive summary of all of the included studies

Author/year	Country	Mean age	Number of patients	Sex (M/F)	Sample size study group	Sample size control group	Intervention control group	Type of laser	Wavelength (nm)	Number of sessions	Treatment time (weeks)	Follow-up (month)	Clinical examination and blood tests	Outcome measurements
Arbabi Kalati et al. 2015 [35]	Iran	46.9	20	-	10	10	Sham laser	Red	630	4	2	-	Yes	NSR, OHIP-14
Spanemberg et al. 2015a [33]	Spain	61.73	58	08/50	19	19	Sham laser	Red	685	9	3	2	Yes	VAS, NRS, OHIP-14
Spanemberg et al. 2015b [33]	Spain	65.5	58	08/50	20		Sham laser	Infrared	830	9	3	2	Yes	VAS, NRS, OHIP-14
Valenzuela et al. 2017a [34]	Spain	65.5	44	03/41	16	12	Sham laser	Infrared	815	4	4	2	-	VAS, OHIP-14, Xerostomia Inventory, HAD, PGI-I
Valenzuela et al. 2017b [34]	Spain	45	44	03/41	16		Sham laser	Infrared	815	4	4	2	-	VAS, OHIP-14, Xerostomia Inventory, HAD, PGI-I
Barbosa et al. 2018 [36]	Brazil	45	15	06/09	10	5	Pharmacotherapy	Red	660	4	4	-	Yes	VAS
Bardellini et al. 2019 [38]	Italy	60.31	85	00/85	43	42	Sham laser	Red – infrared	660–970	10	10	-	-	VAS, OHIP-14, QLROH
Škrinjar et al. 2020 [8]	Croatia	61.5	23	03/20	12	11	Sham laser	Red	685	10	2	-	Yes	VAS
de Pedro et al. 2020 [37]	Spain	63.95	20	04/16	20	20	Sham laser	Infrared	810	10	5	-	Yes	VAS, SF‐36, OHIP‐14, Epworth, SCL 90‐R and McGill

*OHIP*-*14* Oral Health Impact Profile-14, *NSR* numeric rating scale, *VAS* visual analogue scale, *HADS* Hospital Anxiety and Depression Scale, *PGI-I* Patient Global Impression of Improvement, *QLROH* quality of life related to oral health, *SF-36* Short Form Health Survey, *SCL 90‐R* Symptom Checklist-90-Revised

### Quality assessment and risk of bias

The quality was assessed using the Jadad scale [31], which consists of five questions, each of which can be assigned either 0 or 1 point, covering three aspects of clinical trials: randomisation, blinding and description of loss in follow-up. The final sum of these points ranges from 0 to 5, and a score of less than 3 determines a high risk of bias. Once again, this analysis was performed independently by each of the two researchers, and if there was any disagreement, the third researcher acted as a mediator.

### Statistical analysis

Statistically significant results from the quantitative analysis have been presented on “forest plot” graphs. In order to attain these results, the variables from comparable studies were collected before being analysed using the Statistical Package for the Social Sciences (SPSS), v. 24.0 (IBM Inc., Madrid, Spain). Results were expressed as mean difference (MD). Heterogeneity was analysed using the *I*^2^ statistic. An *I*^2^ value < 50% and *p* > 0.1 indicate low heterogeneity, so a fixed effect model was performed. However, when *I*^2^ > 50% and *p* < 0.1 indicate considerable heterogeneity, a random effect model was performed [32].

## Results

### Characteristics of the included studies

A total of 103 publications were obtained in the initial searches, 6 of which met the pre-specified criteria for inclusion with regard to pain assessment, and 5 which included the OHIP-14 quality of life questionnaire. Two of the publications were segregated into two independent studies (a and b) given that different laser frequencies had been used with the same protocol [33], and different protocols had been with the same laser frequency [34] in each of the study groups, compared to the same control group. A detailed description of the included studies has been provided in the study selection flow chart (Table 2).

The evaluated interventions included low-level laser therapy as a therapeutic strategy in the treatment group. The following modalities of PBM use were analysed: 630–685 nm [8, 35, 36], 810–830 nm [34, 37], which corresponded to red and infrared light respectively, or both [33, 38]. In terms of the control group, laser off was present in six of the studies [8, 33–35, 37, 38], and the drug, ALA [36], was applied in one study. General information, as well as the additional methods used to assess the adjuvant effects of therapy, have been summarised in Table 2.

### Quality of the studies included

The quality of the studies was evaluated was using the Jadad scale, with all studies scoring greater than 3 (Table 3).

**Table 3:** Classification of the assessment of study quality according to the Jadad scale. All the studies have total score more than 3, determineted low risk of bias in according to Jadad scale classification. [31]

	Randomised	Random described appropriated	Patient blind	Observer blind	Withdrawals handled	Total score
Arbabi Kalati et al. [35]	**1**	**0**	**1**	**1**	**0**	**3**
Spanemberg et al. [33]	**1**	**0**	**1**	**0**	**1**	**3**
Valenzuela et al. [34]	**1**	**1**	**1**	**0**	**1**	**4**
Barbosa et al. [36]	**1**	**0**	**0**	**1**	**1**	**3**
Bardellini et al. [38]	**1**	**1**	**1**	**1**	**1**	**5**
Škrinjar et al. [8]	**1**	**1**	**1**	**1**	**0**	**4**
de Pedro et al. [37]	**1**	**1**	**1**	**1**	**0**	**4**

### Meta-analysis

#### Treatment points

**Fig. 2:**
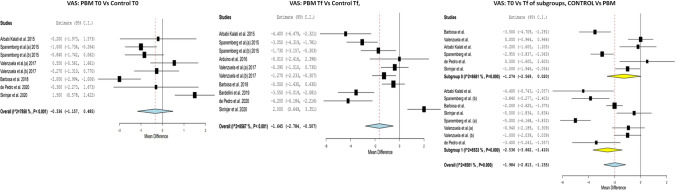
VAS, PBM T0 vs control T0: With regard to pain at T0, no significant differences were found amongst the different study groups. MD =  − 0.336 (IC 95% − 1.157 to 0.485; *p* = 0.423; *I*^2^ = 78.58%, *p* < 0.001). VAS, PBM Tf vs control Tf: When comparing the degree of pain at the final time point (Tf) between the two groups, significant differences were observed, with a greater decrease in pain observed for the PBM group with a MD =  − 1.645 (IC 95% − 2.784 to − 0.507; *I*^2^ = 85.67%, *p* < 0.001). VAS, T0 vs Tf of subgroups, control vs PBM: There was a reduction in the degree of pain in the PBM group MD =  − 2.536 (IC 95% − 3.662 to − 1.410; *I*^2^ = 85.33%, *p* < 0.001) and in the MD control group − 1.274 (− 2.569 to 0.020; *I*^2^ = 86.81%, *p* = 0.054)

With regard to pain at T0, no significant differences were found amongst the different study groups. MD =  − 0.336 (IC 95% − 1.157 to 0.485; *p* = 0.423; *I*^2^ = 78.58%, *p* < 0.001). When comparing the degree of pain at the final time point (Tf) between the two groups, significant differences were observed, with a greater decrease in pain observed for the PBM group with a MD =  − 1.645 (IC 95% − 2.784 to − 0.507; *I*^2^ = 85.67%, *p* < 0.001). In addition, when we compared the Tf and the initial/baseline (T0), there was a reduction in the degree of pain in the PBM group MD =  − 2.536 (IC 95% − 3.662 to − 1.410; *I*^2^ = 85.33%, *p* < 0.001) and in the MD control group − 1.274 (− 2.569 to 0.020; *I*^2^ = 86.81%, *p* = 0.054) (Fig. 2).

**Fig. 3:**
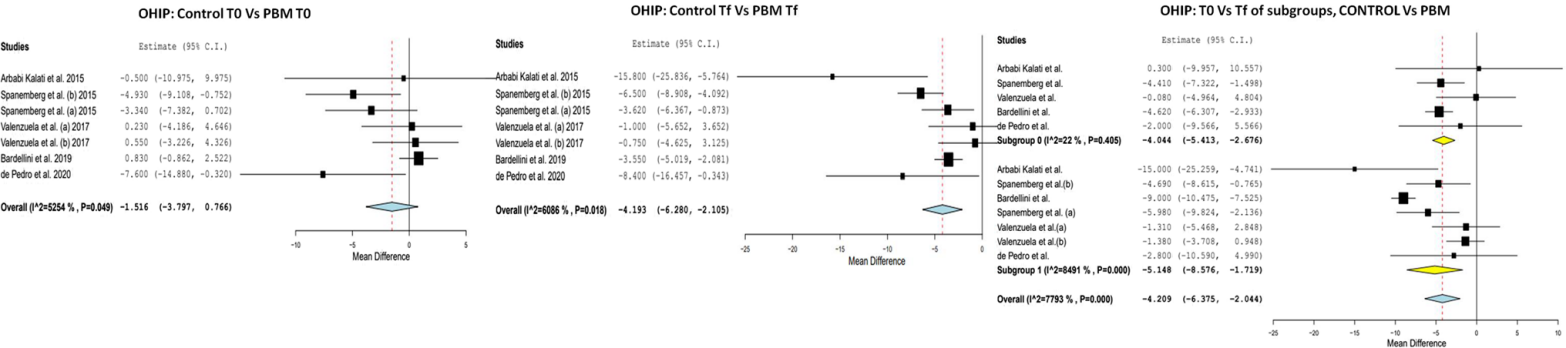
OHIP, PBM T0 vs control T0: When evaluating the degree of pain and the OHRQL at time T0, no significant differences were found between the study and control groups MD =  − 1.516 (IC 95% − 3.797 to 0.485; *p* = 0.766; *I*^2^ = 52.54%, *p* < 0.193). OHIP, PBM Tf vs control Tf: When comparing the OHRQL at Tf between the two groups, significant differences were observed, with a greater decrease in the PBM group with a MD =  − 4.193 (IC 95% − 6.280 to − 2.105; *I*^2^ 60.86%, *p* < 0.001). OHIP, T0 vs Tf of subgroups, control vs PBM: an improvement in OHRQL was observed in both groups, more significant in the PBM group MD =  − 5.148 (IC 95% − 8.576 to − 1.719; *I*^2^ = 84.91%, *p* = 0.003) than in the control group, MD − 4.044 (− 5.413 to − 2.676; *I*^2^ = 22%, *p* < 0.001)

When evaluating the degree of the OHRQL at time T0, no significant differences were found between the study and control groups MD =  − 1.516 (IC 95% − 3.797 to 0.485; *p* = 0.766; *I*^2^ = 52.54%, *p* < 0.193). When comparing the OHRQL at Tf between the two groups, significant differences were observed, with a greater decrease in the PBM group with a MD =  − 4.193 (IC 95% − 6.280 to − 2.105; *I*^2^ 60.86%, *p* < 0.001). Furthermore, when comparing the Tf and the T0, an improvement in OHRQL was observed in both groups, although this was more significant in the PBM group MD =  − 5.148 (IC 95% − 8.576 to − 1.719; *I*^2^ = 84.91%, *p* = 0.003) than in the control group, MD − 4.044 (− 5.413 to − 2.676; *I*^2^ = 22%, *p* < 0.001) (Fig. 3).

### Laser modality

**Fig. 4:**
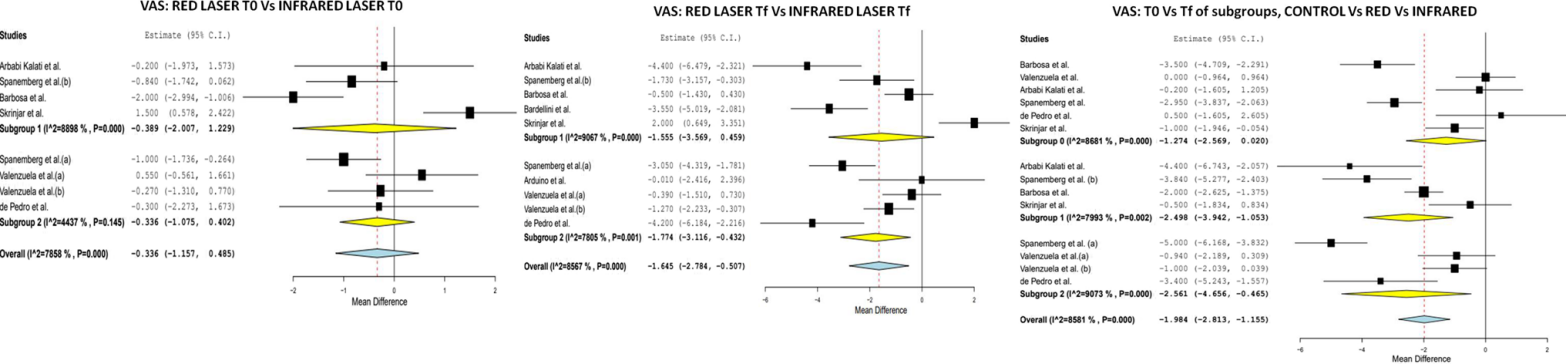
VAS, red laser T0 vs infrared laser T0: With regard to the laser modality used (red/infrared), in terms of pain at T0, no differences were found between the red laser group and the control group, MD =  − 0.389 (IC 95% − 2.007 to 1.229; *I*^2^ = 88.98%, *p* = 0.638), nor between the infrared laser group and the control group, MD =  − 0.336 (IC 95% − 1.075 to 0.402; *I*^2^ = 44.37%, *p* = 0.372). VAS, red laser Tf vs infrared laser Tf: At Tf, no significant differences were found between the red laser group and the control group, MD =  − 1.555 (IC 95% − 3.569 to 0.459; *I*^2^ = 90.67%, *p* = 0.130); however, some differences were found between the infrared modality and the control group, MD =  − 1.774 (IC 95% − 3.116 to − 0.432; *I*^2^ = 78.05%, *p* = 0.010). VAS, T0 vs Tf of subgroups, control vs red vs infrared: an improvement in pain was observed, MD =  − 2.498 (IC 95% − 3.942 to − 1.053; *I*^2^ = 79.93%, *p* < 0.001), and this was higher when infrared laser was used, with a MD =  − 2.561 (IC 95% − 4.656 to − 0.465; *I*^2^ = 90.73%, *p* = 0.017)

With regard to the laser modality used (red/infrared), in terms of pain at T0, no differences were found between the red laser group and the control group, MD =  − 0.389 (IC 95% − 2.007 to 1.229; *I*^2^ = 88.98%, *p* = 0.638), nor between the infrared laser group and the control group, MD =  − 0.336 (IC 95% − 1.075 to 0.402; *I*^2^ = 44.37%, *p* = 0.372). At Tf, no significant differences were found between the red laser group and the control group, MD =  − 1.555 (IC 95% − 3.569 to 0.459; *I*^2^ = 90.67%, *p* = 0.130); however, some differences were found between the infrared modality and the control group, MD =  − 1.774 (IC 95% − 3.116 to − 0.432; *I*^2^ = 78.05%, *p* = 0.010). Furthermore, when we compared the Tf and the initial T0 for the red laser, an improvement in pain was observed, MD =  − 2.498 (IC 95% − 3.942 to − 1.053; *I*^2^ = 79.93%, *p* < 0.001), and this was higher when infrared laser was used, with a MD =  − 2.561 (IC 95% − 4.656 to − 0.465; *I*^2^ = 90.73%, *p* = 0.017) (Fig. 4).

**Fig. 5:**
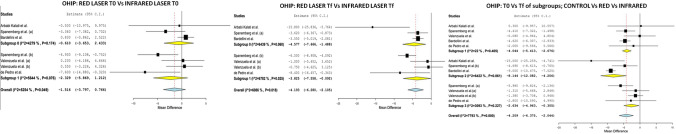
OHIP, red laser T0 vs infrared laser T0: With regard to the quality of life at T0, no differences were found between the red laser group and the infrared laser group, MD =  − 0.610 (IC 95% − 3.653 to 2.433; *I*^2^ = 42.79%, *p* = 0.694) and MD =  − 2.329 (IC 95% − 5.869 to 1.212; *I*^2^ = 56.44%, *p* = 0.197), respectively. OHIP laser Tf vs infrared laser Tf: At Tf, significant differences were found between the two groups, with a higher MD in the red laser group, MD =  − 4.577 (IC 95% − 7.666 to − 1.488; *I*^2^ = 64.39%, *p* = 0.004) than in the infrared group, MD =  − 3.825 (IC 95% − 7.558 to − 0.092; *I*^2^ = 67.82%, *p* = 0.045). OHIP, T0 vs Tf of subgroups, control vs red vs infrared: a greater improvement was observed in terms of quality of life for MD =  − 8.144 (IC 95% − 12.082 to − 4.206; *I*^2^ = 64.22%, *p* = 0.027), compared to infrared MD =  − 2.634 (IC 95% − 4.963 to − 0.305; *I*^2^ = 30.93%, *p* < 0.001)

With regard to the quality of life at T0, no differences were found between the red laser group and the infrared laser group, MD =  − 0.610 (IC 95% − 3.653 to 2.433; *I*^2^ = 42.79%, *p* = 0.694) and MD =  − 2.329 (IC 95% − 5.869 to 1.212; *I*^2^ = 56.44%, *p* = 0.197), respectively. At Tf, significant differences were found between the two groups, with a higher MD in the red laser group, MD =  − 4.577 (IC 95% − 7.666 to − 1.488; *I*^2^ = 64.39%, *p* = 0.004) than in the infrared group, MD =  − 3.825 (IC 95% − 7.558 to − 0.092; *I*^2^ = 67.82%, *p* = 0.045). When we compared the Tf and T0 for the red laser, a greater improvement was observed in terms of quality of life for MD =  − 8.144 (IC 95% − 12.082 to − 4.206; *I*^2^ = 64.22%, *p* = 0.027), compared to infrared MD =  − 2.634 (IC 95% − 4.963 to − 0.305; *I*^2^ = 30.93%, *p* < 0.001) (Fig. 5).

## Discussion

Currently, the basic therapeutic strategies for treating BMS focus on attempting to reduce pain and improve OHRQL by reducing xerostomia, stress level and anxiety [1]. The assessed studies measured pain and OHRQL using the VAS, or the NVS (0–10) and the OHIP-14 test, respectively.

The data obtained revealed soft laser as an important tool for managing BMS, and it was possible to prove its effectiveness, both in terms of reducing pain and in improving OHRQL, predominantly in the infrared modality.

### Assessment of patient-perceived pain

Studies comparing the efficacy of red laser to ALA [36] and infrared laser and clonazepam [39] have presented similar outcomes for both treatments; however, the therapeutic outcomes for laser are slightly better and do not present any adverse effects. However, they also highlight the need for more randomised controlled trials to be conducted, which should be larger and include placebo-controlled therapeutic approaches.

The study by Valenzuela et al. [34] included three groups: group I and group II with active laser in infrared mode, and group III with laser off. The scores obtained from the patients treated with PBM showed a significant decrease in the severity of the burning sensation from the offset, while the control group did not report any significant differences at any time during the evaluation. No significant differences were reported between groups l and ll that received different doses of PBM. Spanemberg et al. [33]*,* divided their population sample into four groups, but for this review, only groups ll, lll and IV, in which infrared, red, and laser off irradiation was used, respectively, with the same frequency and number of sessions, were considered. At the end of the treatment, the symptoms in all of the groups had decreased; however, this decrease differed significantly in the infrared group compared to the control group, and no significant differences were recorded between groups lll and lV, the red and off laser groups, respectively. Antonić et al.’s study published in 2017 also reported that the use of infrared laser was more effective than the use of red laser [40].

In a single study, Bardellini et al. [38] used a laser device with irradiation in the combined red and infrared wavelengths and reported that after the full course of therapy, patients treated with PBM reported a significant decrease in symptoms, which was maintained at the one-month follow-up, thus supporting the use of PBM for treating BMS.

The studies conducted by both Arbabi et al. [35] and Spanenberg et al. [33] showed that the use of PBM significantly decreases the burning sensation in patients’ suffering from BMS. A total of 100% of patients in de Pedro et al.’s study [37] experienced less pain at the end of treatment, an improvement that was maintained at the one-month follow-up, and which remained at 90% at the 4-month follow-up, with no variation in the control group. Contrary to these results, Škrinjar et al. [8] reported that all patients reported fewer burning symptoms after therapy, regardless as to whether their group underwent PBM or whether they formed part of the control group.

### Evaluation of OHRQL, OHIP-14

De Pedro et al. [37] reported that the OHIP-14 scores decreased in the study group and increased in the control group when comparing the initial and final times; however, no significant differences between the two group were recorded. According to Bardellini et al. [38] and Arbabi et al. [35], the use of PBM was associated with an improvement (decrease) in the score and statistically significant differences existed between the treatment and control groups.

According to Valenzuela et al. [34], the groups of patients treated with PBM already showed a significant decrease at two weeks of treatment, a result which stabilised when comparing at the 2nd and 4th weeks, the weeks corresponding to the middle and end of treatment. No significant differences were observed between two groups that received different doses of PBM, and patients in the control group did not show significant differences at any of the evaluated time points.

In the study by Spanemberg et al. [33], there was a significant decrease in scores in both the PBM and control groups when comparing the assessment at beginning and at the end of treatment, and this was maintained at the eight-week follow-up. The difference was most significant in the infrared laser group, with no significant differences reported in the red laser and control groups.

Other studies that were found, but which were not included in the meta-analysis as they did not meet the inclusion criteria, also confirmed the role of laser in improving BMS. (Table 4) [26, 41–43].

**Table 4:** Studies excluded from the review and meta-analysis

Author/year	Country	Type of study	Reason for exclusion
Pellegrini et al. 2010	Brazil	Doctoral thesis	Type of study
Alfaya et al. 2010	Brazil	Case report	Type of study/there is no control group
Romeo et al. 2010	Italy	Pilot study	Type of study/there is no control group
Kato et a. 2010	Brazil	Pilot study	Type of study/there is no control group
Yang et al. 2011	Taiwan	Case series	Type of study/there is no control group
dos Santos et al. 2011	Brazil	Case series	Type of study/there is no control group
Vukoja et al. 2011	Croatia	Letter to the editor	Type of study
Pezelj-Ribarić et al. 2012	Croatia	Randomised controlled trial	The article only presented the VAS values for the study group, and the authors did not return our attempts to contact them. No OHIP rating
Brailo et al. 2013	Croatia	Pilot study	Type of study/there is no control group
dos Santos et al. 2015	Brazil	Prospective clinical study reports	There is no control group
Sugaya et al. 2016	Brazil	Randomised controlled trial	Reported the VAS scale from 0 to 5. No OHIP rating
Arduino et al. 2016	Italy	Pilot study	Reported the VAS scale from 0 to 100 and OHIP-49
Antonić et al. 2017	Croatia	Case series	Type of study/there is no control group
Cui et al. 2017	China	Randomised controlled trial	Article has no DOI; we did not have access to the full text
Sikora et al. 2018	Croatia	Randomised controlled trial	It only presented the comparative value of the initial and final VAS scale and OHIP. We tried to contact the authors, but they did not reply
Spanemberg et al. 2019	Brazil	Randomised controlled trial	Does not provide detailed Mean and SD starting and ending values to include in the graphs. The author replied to our emails, but did not have access to. No OHIP rating

Recently published systematic reviews on PBM [44, 45] include articles that were excluded from our study because they used pain scales with different scores [42], because they did not have a control group, or because the data provided was insufficient to be included in a meta-analysis [42, 46]. In this way, and after verifying the powerful biases of these studies, the present study was justified.

The diagnosis and treatment of BMS is still not very clear, as it is considered a multifactorial condition, with neuropathic, endocrinological, and psychological components [4]. This is why it is so important for a detailed medical history, which covers both general and oral health to be taken, which is usually followed by a set of ancillary investigations including a full blood count, serum iron determination, vitamin B12, folate and blood glucose levels. In addition, the patients’ knowledge of the chronic nature of the disease and the general absence of a history of malignancy is essential in order to limit anxiety and the risk of developing cancerophobia [1, 47].

Limitations of this study included the different final assessment time points (Tf ranging from 2 to 10 weeks), short follow-up periods, the relatively low number of participants and the high variability in the metrics used to assess outcomes with heterogeneous study designs. These limitations have been minimised thanks to a comprehensive design, very strict inclusion criteria and the thorough evaluation of the data provided.

## Conclusion

The management of patients with BMS is difficult and often frustrating. The correct diagnosis of this syndrome and the exclusion of local or systemic factors that may be associated with burning mouth symptoms are essential, and, likewise, it is important to continue to search for new therapeutic alternatives. Although various treatment modalities have been proposed, such as pharmacological intervention, behavioural therapy and psychotherapy, there is no definitive treatment that always proves effective for the majority of patients with BMS. This systematic review and meta-analysis has concluded that amongst the different laser protocols, the ones in which red laser were used were statistically more effective in reducing BMS symptoms, in contrast to the results of studies in which red and infrared radiation were compared. Likewise, PBM resulted in a clear improvement in OHRQL compared to other treatment modalities. Further prospective studies with adequate epidemiological designs are required in order to better understand the relationship that exists between BMS and psychological and neuropathic factors.
